# Clinical Value of Detecting Fecal Calprotectin by Using Colloidal Gold Assay in Screening or Diagnosing Crohn's Disease

**DOI:** 10.1155/2023/8866828

**Published:** 2023-11-09

**Authors:** Wangdong Zhang, Yanyun Fan, Meijun Chen

**Affiliations:** ^1^Center of Clinical Laboratory, Zhongshan Hospital, School of Medicine, Xiamen University, Xiamen 361004, China; ^2^Department of Gastroenterology, Zhongshan Hospital, School of Medicine, Xiamen University, Xiamen 361004, China

## Abstract

**Background:**

Crohn's disease (CD) is a chronic inflammatory disease, and its incidence is gradually increasing. Thus, the use of a simple and convenient examination method to detect CD in the natural population as early as possible is crucial. This study is aimed at using the colloidal gold semiquantitative assay to detect fecal calprotectin (FCP) and determine whether it is helpful in screening or diagnosing CD.

**Methods:**

Using a prospectively maintained database, 59 patients with CD were analyzed using FCP measurement. Subsequently, 76 patients and 89 healthy individuals were assigned to the gastrointestinal dysfunction and control groups, respectively. To aid in the screening or diagnosis of CD, the receiver operating characteristic curve was used to determine the diagnostic efficacy of FCP thresholds. Sensitivity, specificity, positive predictive value (PPV), and negative predictive value (NPV) were presented with 95% confidence intervals (CIs).

**Results:**

Patients with CD showed significantly higher FCP levels. Compared with the healthy population, when the FCP level cut-off was 15 *μ*g/g and 60 *μ*g/g, the sensitivity, specificity, PPV, and NPV for CD diagnosis were 98.3% (CI, 95.0%–100%) and 78.0% (CI, 67.4–88.6%), 84.3% (CI, 76.7%–91.8%) and 98.9% (CI, 96.7%–100%), 80.6% (CI, 71.5%–89.7%) and 97.9% (CI, 93.7%–100%), and 98.7% (CI, 96.2%–100%) and 87.1% (CI, 80.6%–93.6%), respectively. The AUCs were 0.969 (CI, 0.941–0.997). Compared with the gastrointestinal dysfunction group, using the same FCP level cut-off, the sensitivity, specificity, PPV, and NPV for CD diagnosis were 98.3% (CI, 95.0%–100%) and 78.0% (CI, 67.4%–88.6%), 71.1% (CI, 60.9%–81.3%) and 89.5% (CI, 82.3%–96.7%), 72.5% (CI, 62.7%–82.3%) and 85.2% (CI, 75.7%–94.7%), and 98.1% (CI, 94.5%–100%) and 84.0% (CI, 76.0%–92.0%), respectively. The AUCs were 0.908 (CI, 0.856–0.960).

**Conclusion:**

Detecting FCP by using the colloidal gold semiquantitative assay can be effective in screening and adjunct diagnosing of CD.

## 1. Introduction

Crohn's disease (CD) is a chronic inflammatory granulomatous disease of the digestive tract of unknown etiology, which involves the digestive tract from the oral cavity to the anus. No gold standard exists for the diagnosis of CD. The diagnostic criteria recommended by the World Health Organization include clinical presentation, radiographic examination, endoscopy, biopsy, and surgical specimens. However, these indicators do not detect CD early. Calprotectin is an inflammatory reactive protein that is stable in feces. Several studies have shown that detecting fecal calprotectin (FCP) can help determine the presence of inflammatory bowel disease [1–4]. One study used retrieved literature to construct an FCP cut-off of 50,100,200 *μ*g/g to analyze the sensitivity and specificity for diagnosing CD [2]. One study examined the relationship between FCP levels and intestinal inflammation in patients with CD through prospective data collection and retrospective cohort analysis. The final recommendation is that more prospective studies are needed to clarify FCP cut-off levels [4]. These studies used enzyme-linked immunosorbent assay or chemical methods to detect FCP, and feces should be stored for a specific time before testing. Therefore, these methods are not suitable for screening CD because of requiring instrumentation and being time-consuming. However, these shortcomings are resolved using a colloidal gold semiquantitative assay for FCP. Thus, we used the colloidal gold semiquantitative method to detect FCP in newly diagnosed patients with CD and compared healthy control and gastrointestinal dysfunction groups. Additionally, we analyzed whether FCP can be used as an auxiliary diagnostic marker of CD and to detect CD in the natural population as early as possible.

## 2. Methods

### 2.1. Patients with CD

From November 2021 to November 2022, 59 patients with CD, of which 40 and 19 were men and women, respectively, were diagnosed at Zhong Shang Hospital in Xiamen. Their age range was 14 years to 64 years, with a median age of 26 years. The diagnostic criteria for confirmed CD were based on the recommendations of the World Gastroenterology Organization [5]. The patients were consulted due to abdominal pain and diarrhea (47 cases), change of defecation habit (5 cases), perihepatic abscess (4 cases), hematochezia (2 cases), and anal fistula (1 case). All patients underwent colonoscopy and computed tomography three-dimensional imaging. Depending on the need of the disease, some patients underwent electronic gastroscopy and pathological biopsy. Moreover, all patients signed an informed consent form. All patients did not take CD drugs before diagnosis.

### 2.2. Gastrointestinal Dysfunction Group

This group consisted of 76 patients, of which 57 and 19 were men and women, respectively, with gastrointestinal dysfunction accompanied by abdominal pain and diarrhea. Their age range was 14–66 years, with a median age of 32 years. All patients visited the outpatient department of Zhongshan Hospital in Xiamen. This group refers to participants fulfilling Roma IV criteria for Diarrhea Predominant-Irritable Bowel Syndrome without a history of doctor-diagnosed organic gastrointestinal diseases or a history of bowel resection.

### 2.3. Healthy Group

The healthy group consisted of 89 healthy individuals, of which 62 and 27 were men and women, respectively, who were examined by a doctor at Zhongshan Hospital in Xiamen, including doctor's inquiries, physical examinations, and laboratory tests, such as blood tests, stool tests, and urine tests. Their age range was 14–62 years, with a median age of 33 years. This group is a population to the hospital for regular health checkups, without any symptoms and abnormal results.

### 2.4. FCP Measurement

The FCP level was assessed routinely by using the colloidal gold semiquantitative assay. Additionally, the strip contained anti-FCP-coated monoclonal antibodies prefixed to the detection region (T) of the chromatographic membrane and goat anti-rabbit IgG antibodies to the control regions (R and C). The labeling pad contains precoated colloidal gold-labeled anti-FCP-labeled monoclonal antibody and colloidal gold-labeled rabbit IgG antibody. When a sample is positive, the FCP in the sample can be mixed with the colloidal gold-labeled anti-FCP-labeled anti-FCP monoclonal antibody to form an immune complex, the composite and sample flow in the direction of the absorbent paper in the nitrocellulose membrane. The complex binds to the coated anti-FCP-coated monoclonal antibody as it passes through the detection region (T) and appears as a red band. The color development intensity was positively correlated with FCP content in the samples. The criteria for determining the adequacy of the specimen and the normality of the chromatographic process, as well as for the internal control of the reagent, are the red bands in the control (R) and quality control (C)-RRB areas. In the control region (R), the FCP content was 60 *μ*g/g. In the detection region (T), the lowest FCP content was 15 *μ*g/g. FCP was tested in patients with suspected CD and in controls who visited Zhongshan Hospital, that is, affiliated with Xiamen University, from November 2021 to November 2022. FCP was considered positive, weakly positive, and negative if the values were >60, 15 < FCP < 60, and <15 *μ*g/g, respectively. FCP measurement to wait 15 minutes can be the result.

### 2.5. Statistical Analysis

IBM SPSS v22.0 software was used to perform the analyses. Based on the statistical distribution, the characteristics at the time of inclusion were expressed as percentages or averages. The *χ* 2 test was used to compare the parameters between two independent groups (i.e., CD and control groups). The receiver operating characteristic (ROC) curve was used to determine the diagnostic efficacy of FCP thresholds to aid in diagnosing CD. Sensitivity, specificity, positive predictive value (PPV), and negative predictive value (NPV) were expressed as 95% CIs for each threshold.

## 3. Results

### 3.1. CD Population Characteristics and FCP Level

This study included 59 patients with CD, including the pure ileum CD group, with colonic or ileocolonic involvement. The control and gastrointestinal dysfunction groups consisted of 89 healthy individuals and 76 patients, respectively.  Table 1 shows the basic patient characteristics and comparison of FCP levels.

### 3.2. Comparison of FCP Levels between Patients with CD and Healthy Individuals

In CD patients, FCP was negative, positive, and weakly positive in 1 patient (1.7%), 46 (78.0%) patients, and 12 (20.3%) patients, respectively. In healthy individuals, FCP was positive, weakly positive, and negative in 1 (1.1%), 13 (14.6%), and 75 patients (84.3%), respectively. If 60 *μ*g/g was the cut-off value, FCP was positive and negative in 46 and 13 patients with CD, whereas it was positive and negative in 1 and 88 healthy individuals, respectively (Table 2). Moreover, the positive rate of FCP between the two groups was statistically significant.

If 15 *μ*g/g was used as the cut-off value, FCP was positive and negative in 58 and 1 patient with CD, whereas it was positive and negative in 14 and 75 healthy individuals, respectively (Table 3). Moreover, the positive rate of FCP between the two groups was statistically significant.

### 3.3. Comparison of FCP between CD and Gastrointestinal Dysfunction Groups

In the gastrointestinal dysfunction group, FCP was positive, weakly positive, and negative in 8 (10.5%), 14 (18.4%), and 54 patients (71.1%), respectively. If 60 *μ*g/g was the cut-off value, FCP was positive and negative in 46 and 13 patients with CD and 8 and 68 patients in the gastrointestinal dysfunction group, respectively (Table 4). Moreover, the positive rate of FCP between the two groups was statistically significant.

If 15 *μ*g/g was used as the cut-off value, FCP was positive and negative in 58 and 1 patient with CD, and 22 and 54 patients in the gastrointestinal dysfunction group, respectively (Table 5). Moreover, the positive rate of FCP between the two groups was statistically significant.

### 3.4. ROC Curves of FCP Values between CD Patients and the Control Group

**Figure 1:**
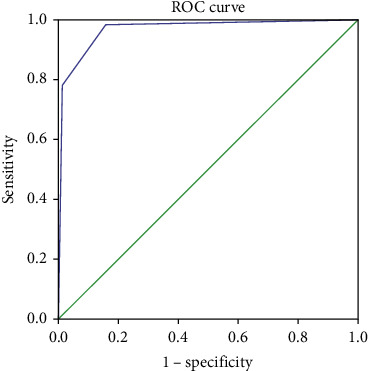
Receiver operating characteristic curves of FCP values between CD patients and healthy individuals.

The sensitivity and specificity of CD diagnosis at different FCP cut-off values between CD patients and healthy individuals were analyzed. When the FCP cut-off values were 60 and 15 *μ*g/g, the sensitivity and specificity of CD diagnosis were 78.0% and 98.9% and 98.3% and 84.3%, respectively. The AUCs were 0.969 (CI, 0.941–0.997) (Figure 1).

**Figure 2:**
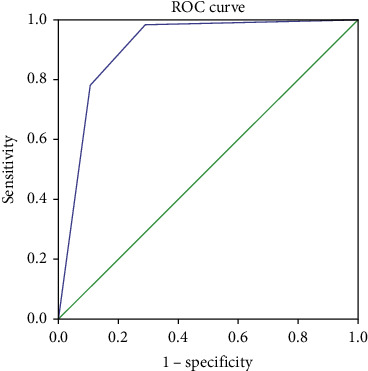
Receiver operating characteristic curves of FCP values between CD patients and the gastrointestinal dysfunction group.

The sensitivity and specificity of CD diagnosis at different FCP cut-off values between CD patients and the gastrointestinal dysfunction group were analyzed. When the FCP cut-off value was 60 and 15 *μ*g/g, the sensitivity and specificity of CD were 78.0% and 89.5% and 98.3% and 71.1%, respectively. The AUCs were 0.908 (CI, 0.856–0.960) (Figure 2).

## 4. Discussion

CD is a chronic intermittent inflammatory bowel disease, with abdominal pain and diarrhea as its common symptoms. In radiologic studies, discontinuous or segmental changes are seen, and endoscopies show a pebble-like appearance or longitudinal ulceration. Additionally, biopsies show noncaseous granulomas or fissures, fistulas, and perianal lesions in some cases. Our results showed that abdominal pain and diarrhea were mainly the initial symptoms of CD. Additionally, changes in defecation habits and perianal lesions also suggest the possibility of CD. The main type of CD was ileocolic based on the location of the lesion. Based on disease behavior, the main types of CD were narrow and nonnarrow nonpenetrating types. Most patients were diagnosed after more than 1 month from symptom onset. Over the course of more than a decade, clinical phenotypic characteristics included a higher prevalence of males, and a higher prevalence of ileocolonic involvement among East Asian CD patients [6], which did not change. Compared with Mak et al.'s report [1], CD patients were younger, and cases of stricturing were more. This indicates a trend towards younger age and more severe disease in CD patients. The pathogenesis of CD is unclear. Some studies have shown that the causes of CD are complex, and genetic factors alone cannot explain most cases [6]. Moreover, environmental factors also play an important role in developing CD, such as smoking [7]. In addition, experimental studies in mice have shown that some dietary nutrients promote intestinal inflammation by modulating gut microbiota [8]. Therefore, early detection of intestinal inflammation is beneficial for further diagnostic evaluation or timely intervention, such as quitting smoking and improving dietary habits; thus, changing the disease development course is worth studying. The preferred methods for intestinal inflammation examination are electronic colonoscopy and biopsy, but it is expensive, not readily available, and sometimes plagued by the small intestinal obstruction; thus, biomarkers have emerged as an inexpensive and simple screening tool.

Calprotectin is a calcium-binding protein heterodimer (including S100A8 and S100A9) that is isolated from neutrophils and linked by a covalent bond between two heavy chains and a light chain. Additionally, S100A8 and S100A9 genes are localized on chromosome 1q21 and are mainly expressed in neutrophils, monocytes, dendritic cells, etc. [9–11]. During inflammation, S100A8 and S100A9 can be specifically induced [12]. Calprotectin is found in body fluids, cells, tissues, and excreta. Furthermore, FCP is readily available and remains stable in feces for at least 3 days at room temperature [13]. Studies have shown that FCP correlates with the number of neutrophils in the intestinal lumen [14, 15]; thus, it could be used to detect intestinal inflammation. FCP is a useful biomarker for detecting intestinal inflammation and diagnosing and monitoring patients with inflammatory bowel disease [2, 16–18]. Shimoyama et al. examined the value of fecal biomarkers in screening for small intestinal inflammation in patients with CD. The sensitivity and specificity were 69% and 82% for disease diagnosis, respectively, for the calprotectin cut-off value at 140 *μ*g/g, and the area under the ROC curve (AUC) was 0.82 [16]. Jung et al. suggested that the optimal diagnostic threshold for FCP was 100 *μ*g/g, with sensitivity and specificity of 73% and 73%, respectively [2]. Stawczyk-Eder et al. compared the diagnostic value of FCP in patients with CD with different disease sites and concluded that FCP could be used as a diagnostic index [17]. Simon et al. systematically analyzed the published literature and found that the sensitivity and specificity of FCP were 42.9%–100% and 66.7%–100% and 50%–100% and 28.6%–100% at small bowel and large bowel, respectively [18]. Compared with the healthy population, our study showed that the sensitivity, specificity, PPV, and NPV for CD diagnosis with a threshold of 60 *μ*g/g for FCP were 78.0%, 98.9%, 97.9%, and 87.1%, respectively, whereas those with a threshold of 15 *μ*g/g for FCP were 98.3%, 84.3%, 80.6%, and 98.7%, respectively. Compared with the gastrointestinal dysfunction group, our study showed that the sensitivity, specificity, PPV, and NPV for disease diagnosis with a threshold of 60 *μ*g/g for FCP were 78.0%, 89.5%, 85.2%, and 84.0%, respectively, whereas those with a threshold of 15 *μ*g/g for FCP were 98.3%, 71.1%, 72.5%, and 98.1%, respectively. Our results suggest that the efficiency of screening and diagnosing CD using a colloidal gold semiquantitative detecting method for fecal calprotectin (FCP) is similar to the quantitative method described in existing literature references[16]. This finding indicates that the colloidal gold semiquantitative method is a viable alternative for detecting FCP levels.

One advantage of using the colloidal gold semiquantitative detection method is its simplicity and speed. It is a straightforward procedure that can provide results quickly. The interpretation of the results is also relatively easy, making it suitable for detecting FCP levels in a family environment, potentially facilitating CD screening.

Moreover, this method is particularly useful in individuals with high-risk factors for CD. These risk factors may include perianal lesions (beyond hemorrhoids), having a first-degree relative with inflammatory bowel disease, experiencing significant weight loss (approximately 5% of body weight) within the past three months, and having abdominal pain persisting for more than three months, nocturnal diarrhea, among others [19]. By using FCP as a screening indicator, healthcare professionals can identify individuals who may have CD.

In summary, the study suggests that FCP is a useful screening indicator for CD, and the colloidal gold semiquantitative detecting method offers a simple, fast, and easily interpretable approach for detecting FCP levels. This method can be particularly helpful in individuals with high-risk factors for CD. However, it is important to note that further research and clinical validation may be necessary to confirm these findings and establish the widespread use of this method in CD screening and diagnosis.

Some patients with CD and in the control group had weakly positive FCP levels. Studies have shown that some patients with CD have FCP levels < 60 *μ*g/g [20]. Because FCP concentrations in healthy adults range from approximately 10 to 50 *μ*g/g, this depends on the study population and assay used [20–22]. Following the kit instructions, the mean level of FCP in healthy individuals was 12 *μ*g/g (95% CI, of 7–18 *μ*g/g). Therefore, the FCP cut-off value for diagnosing CD should be 60 *μ*g/g. Thus, the AUC was 0.969 and 0.908 in patients with CD patients when compared with the healthy population and gastrointestinal dysfunction group, respectively. However, this study has some limitations. First, the number of cases is relatively small. Second, when FCP content exceeds 2000 *μ*g/g, the color development intensity is weakened. All of these can cause results to skew. For example, this article presents a case involving a 19-year-old male patient diagnosed with CD who tested negative for FCP. The patient exhibits the ileocolic type and stricturing type of CD. The cause of this result is unclear. When FCP > 2000 *μ*g/g, the color weakening can be resolved by diluting the sample. In fact, the case of FCP > 2000 *μ*g/g is rare, and its symptoms and signs are obvious even when present. Therefore, quantitative testing is recommended for patients with severe suspicion of CD. However, in FCP weak positive patients, FCP should be detected continuously. As the disease progresses, FCP levels increase in patients with CD. Although the colloidal gold method is simple, it is not possible to obtain an accurate FCP level. Therefore, when the FCP level is positive, the quantitative method should be used to determine the FCP level as far as possible.

In conclusion, FCP as an adjunct diagnostic marker of CD is feasible. Although FCP can be used to detect inflammation in the gut, it cannot distinguish between inflammation causes. Therefore, when FCP results suggest the possibility of CD, individuals should seek medical advice for prompt diagnosis and treatment. In one word, detecting FCP by using the colloidal gold semiquantitative assay can be effective in screening and adjunct diagnosing of CD.

## Figures and Tables

**Table 1:** Baseline characteristics of patients with CD and the control group.

	CD patients (*N* = 59)	Gastrointestinal dysfunction group (*N* = 76)	Healthy individuals (*N* = 89)
Age in years	30.4 ± 12.0	34.0 ± 12.9	34.9 ± 9.2

Male (%)	40 (67.8%)	57 (75%)	62 (69.7%)

Montreal classification	CD (location)		
	L1 (ileum): 5 (8.5%)		
	L2 (colon): 5 (8.5%)		
	L3 (ileocolon): 43 (72.9%)		
	L3 + L4 (upper gastrointestinal): 4 (6.7%)		
	L1 + L4: 2 (3.4%)		
	CD (behavior)		
	B1 (inflammatory): 24 (40.7%)		
	B2 (stricturing): 30 (50.8%)		
	B3 (fistulating): 5 (8.5%)		

FCP (*μ*g/g)	>60 (*μ*g/g): 46 (78.0%)	>60 (*μ*g/g): 8 (10.5%)	>60 (*μ*g/g): 1 (1.1%)
	15-60 (*μ*g/g): 12 (20.3%)	15-60 (*μ*g/g): 14 (18.4%)	15-60 (*μ*g/g): 13 (14.6%)
	<15 (*μ*g/g): 1 (1.7%)	<15(*μ*g/g): 54 (71.1%)	<15 (*μ*g/g): 75 (84.3%)

Note: the age shown is mean ± SD.

**Table 2:** Comparison of FCP levels between patients with CD and healthy individuals (cutoff level = 60 *μ*g/g).

	*n*	Positive	Negative	
CD	59	46	13	
Healthy individuals	89	1	88	*p* < 0.001

**Table 3:** Comparison of FCP levels between patients with CD and healthy individuals (cutoff level = 15 *μ*g/g).

	*n*	Positive	Negative	
CD	59	58	1	
Healthy individuals	89	14	75	*p* < 0.001

**Table 4:** Comparison of FCP levels between patients with CD and gastrointestinal dysfunction group (cutoff level = 60 *μ*g/g).

	*n*	Positive	Negative	
CD	59	46	13	
Gastrointestinal dysfunction group	76	8	68	*p* < 0.001

**Table 5:** Comparison of FCP levels between patients with CD and gastrointestinal dysfunction group (cutoff level = 15 *μ*g/g).

	*n*	Positive	Negative	
CD	59	58	1	
Gastrointestinal dysfunction group	76	22	54	*p* < 0.001

## Data Availability

The raw data supporting the conclusions of this article will be made available by the corresponding author Wangdong Zhang (zwdzwd2021@qq.com), without undue reservation, due to restrictions on hospital management in Zhongshan Hospital, Xiamen University. The case information is stored in the Hospital Information System, and the experimental data are stored in the hospital's Laboratory Information Management System.
